# Supplementary material for: Hydrogel Fluorescence Microsensor with Fluorescence Recovery for Prolonged Stable Temperature Measurements
Source: Sensors (Basel). 2019 Nov 29;19(23):5247. doi: 10.3390/s19235247 (PMC6928776; doi:10.3390/s19235247)
Supplement: Supplementary file 1 [file sensors-19-05247-s001.pdf]

sensors

Electronic Supplementary Information

Hydrogel Fluorescence Microsensor with Fluorescence Recovery for Prolonged Stable Temperature Measurements

Hairulazwan Hashim[1,2,*], Hisataka Maruyama[1], Yusuke Akita[1] and Fumihito Arai[1]

1 Department of Micro-Nano Mechanical Science and Engineering, Nagoya University, Furo-cho, Chikusa-ku, Nagoya 464-8603, Japan; hisataka@mech.nagoya-u.ac.jp (H.M.); akita@biorobotics.mech.nagoya-u.ac.jp (Y.A.); arai@mech.nagoya-u.ac.jp (F.A.)
2 Faculty of Engineering Technology, Universiti Tun Hussein Onn Malaysia, 86400 Parit Raja, Batu Pahat, Johor, Malaysia
* Correspondence: hashim@biorobotics.mech.nagoya-u.ac.jp or azwan@uthm.edu.my (H.H.); Tel.: +81-52-789-5026; Fax: +81-52-789-5104

Received: 19 October 2019; Accepted: 26 November 2019; Published: date

1. Fabrication of the Hydrogel Fluorescence Microsensor

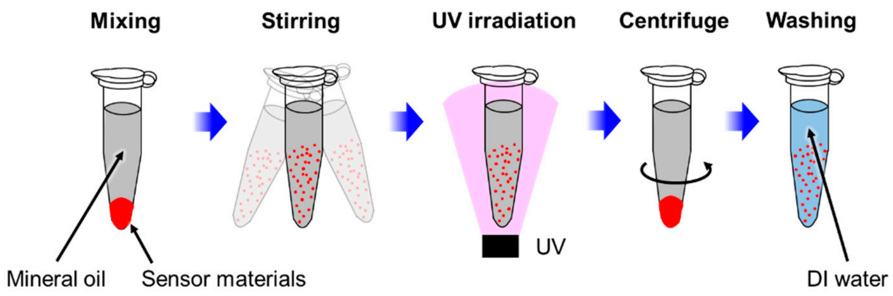

(a)

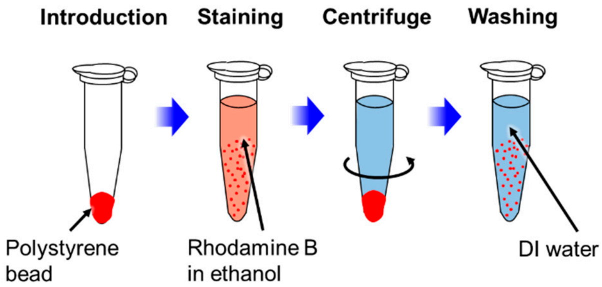

(b)

Figure S1. Fabrication processes for fluorescence microsensors. (**a**) Hydrogel fluorescence microsensor; (**b**) Polystyrene fluorescence microsensor.

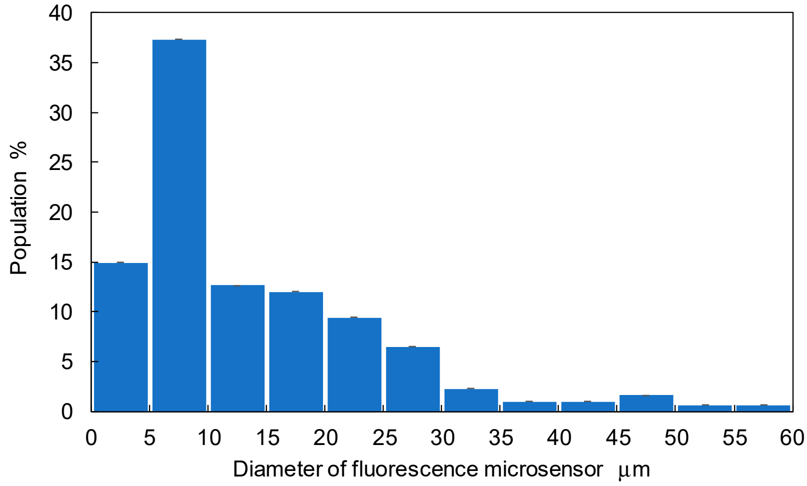

Figure S2. Diameter distribution of the hydrogel fluorescence microsensors (9% PEGDA575).

2. Temperature Measurement Using Hydrogel Fluorescence Microsensors

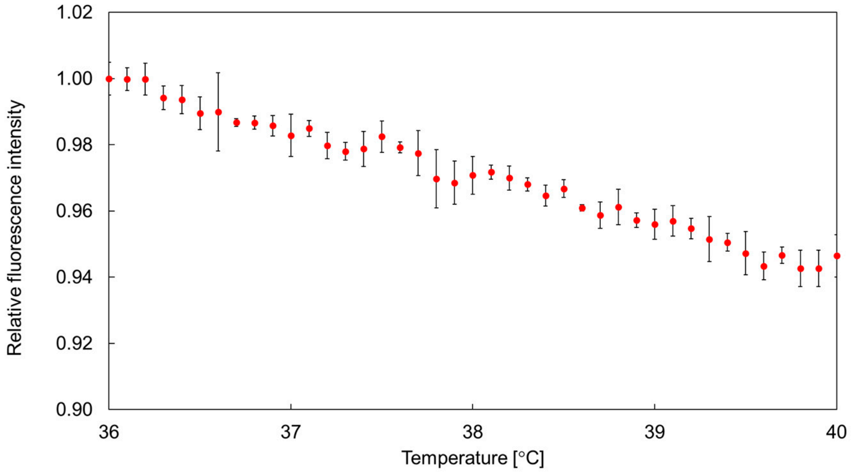

Figure S3. Calibration of the relative fluorescence intensity with temperature.

3. Evaluation of Photobleaching Compensation Using Fluorescent Dyes Other than Rhodamine B

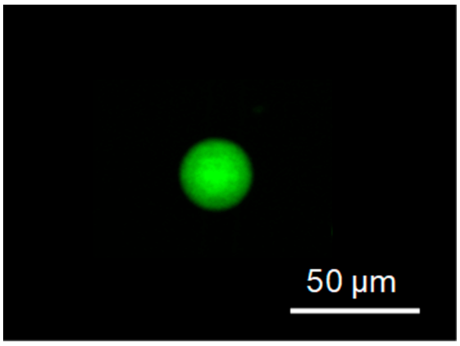

(a)

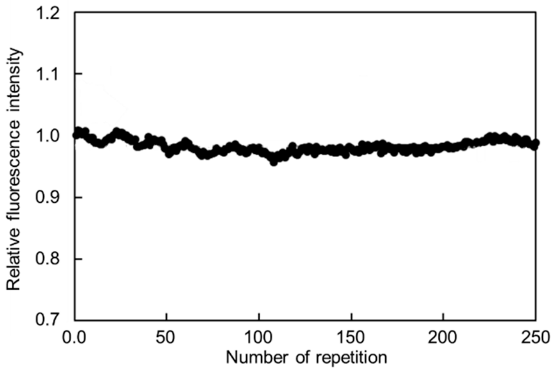

(b)

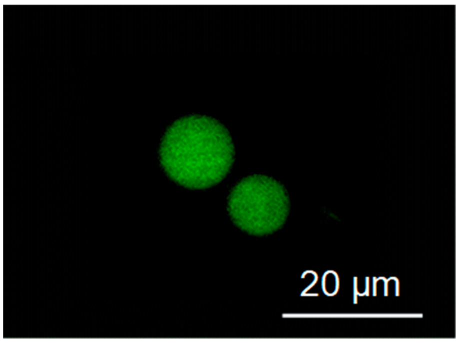

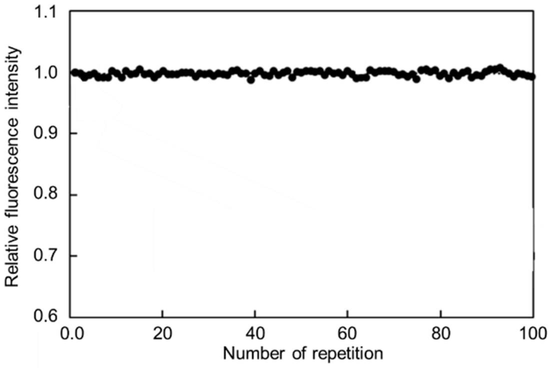

(c) (d)

Figure S4. Photobleaching compensation of hydrogel fluorescence microsensors containing FITC and ($Ru(bpy)_2Cl_2$). (**a**) Fluorescence image of hydrogel fluorescence microsensor containing FITC; (**b**) Photobleaching compensation of hydrogel fluorescence microsensor containing FITC; (**c**) Fluorescence image of hydrogel fluorescence microsensor containing ($Ru(bpy)_2Cl_2$); (**d**) Photobleaching compensation of hydrogel fluorescence microsensor containing ($Ru(bpy)_2Cl_2$).

© 2019 by the authors. Submitted for possible open access publication under the terms and conditions of the Creative Commons Attribution (CC BY) license (http://creativecommons.org/licenses/by/4.0/).
